# Transforming environmental governance: critical action intellectuals and their praxis in the field

**DOI:** 10.1007/s11625-022-01108-z

**Published:** 2022-02-22

**Authors:** Hemant Ojha, Andrea J. Nightingale, Noémi Gonda, Benard Oula Muok, Siri Eriksen, Dil Khatri, Dinesh Paudel

**Affiliations:** 1 University of Canberra, Canberra, Australia; 2 University of Oslo, Oslo, Norway; 3 Swedish University of Agricultural Sciences, Uppsala, Sweden; 4 Jaramogi Oginga Odinga University of Science and Technology, Bondo, Kenya; 5 Norwegian University of Life Sciences, As, Norway; 6 Southasia Institute of Advanced Studies, Kathmandu, Nepal; 7 Appalachian State University, Boone, NC USA

**Keywords:** Transformation, Environmental governance, Critical action intellectuals, Praxis, Social field

## Abstract

Over the past decade, widespread concern has emerged over how environmental governance can be transformed to avoid impending catastrophes such as climate change, biodiversity loss, and livelihood insecurity. A variety of approaches have emerged, focusing on either politics, technological breakthrough, social movements, or macro-economic processes as the main drivers of change. In contrast, this paper presents theoretical insights about how systemic change in environmental governance can be triggered by critical and intellectually grounded social actors in specific contexts of environment and development. Conceptualising such actors as critical action intellectuals (CAI), we analyze how CAI emerge in specific socio-environmental contexts and contribute to systemic change in governance. CAI trigger transformative change by shifting policy discourse, generating alternative evidence, and challenging dominant policy assumptions, whilst aiming to empower marginalized groups. While CAI do not work in a vacuum, nor are the sole force in transformation, we nevertheless show that the praxis of CAI within fields of environmental governance has the potential to trigger transformation. We illustrate this through three cases of natural resource governance in Nepal, Nicaragua and Guatemala, and Kenya, where the authors themselves have engaged as CAI. We contribute to theorising the ‘how’ of transformation by showing the ways CAI praxis reshape fields of governance and catalyze transformation, distinct from, and at times complementary to, other dominant drivers such as social movements, macroeconomic processes or technological breakthroughs.

## Introduction

It is now well accepted that the climate crisis, biodiversity loss, social inequalities, and livelihood insecurity cannot be tackled without systemic change in how environment and natural resources are governed (O’Brien 2012; Scoones et al. 2015; Leichenko and O'Brien 2019; Ely 2021). Three major global agreements signed in 2015—the Paris Climate Agreement, Sustainable Development Goals, and the Sendai Framework for Disaster Risks Reduction—all call for significant changes in development and environmental governance. Furthermore, the COVID-19 pandemic has laid bare the shortcomings of governance structures and systems, reinforcing the need to understand possibilities for transformation (Weible et al. 2020; Schipper et al. 2021). Nevertheless, change is too slow. Transformative change cannot be achieved through incremental approaches; more fundamental shifts in practices, behaviours, and systems are required (Stirling 2014; Blythe et al. 2018). We call such shifts ‘transformation’, which can lead to improved governance and outcomes for society and the environment (O’Brien 2012; Scoones et al. 2020; Chaffin et al. 2016).

Studies have linked the prospects for transformational change to drivers including: globalisation (Bierman and Pattberg 2008), politics and contestations (e.g. Karriem 2009; Kashwan 2017), agency of specific actors (e.g. Stuart et al. 2020; Otto et al. 2020), social movements (e.g. Smith et al. 2016), multi-faceted socio-technical transitions (e.g. Geels and Kemp 2006; Geels 2019), and crises in social-ecological systems (e.g. Butzer 2012; Chaffin et al. 2016). Despite this surge in research, conceptual wisdom on the ‘how’ of transformation remains patchy (Fazey et al. 2018a, b). Complicating matters, changes triggered by these different drivers have not necessarily led to positive outcomes, partly because the project of transformation itself carries with it the risk of technical fixes instead of embracing the contested politics and contextually embedded nature of social change (Blythe et al. 2018; Nightingale et al 2020).

This paper builds from the premise that the interface between the politics of knowledge and human agency is critical for theorizing how transformation takes place in environmental governance. We begin from an analysis of three aspects of this interface. First, a recognition of and willingness to challenge the hegemony of dominant knowledge systems paradigms and practices. Second, the role of human agency in triggering shifts in paradigms and knowledge systems (Otto et al. 2020; Vogel and O’Brien 2021). Third, deconstructing rational policy approaches to solve complex (‘wicked’) environmental issues (Fischer 2003; Wright and Shore 2011). From these starting points, we embrace a ‘political epistemology’ to both understand *and* contribute to changing the problematic state of environmental governance (Strassheim 2015). In particular, our purpose here is to explore the role of knowledge actors, including what they do and how they interact with other actors in transformation.

At the core of our analysis is what we call, critical action intellectuals (CAI), people who contribute to systemic change though their intellectual work and political engagement (praxis) in relation to fields of environmental governance. Our previous work has shown that critical action research that challenges hegemonic knowledge, and experimental actions to demonstrate alternatives, can promote lasting change (Ojha 2013). This kind of work differs from activism and academic research. CAI are different from activists as they take knowledge creation and mobilisation as the primary vehicle for change. Unlike most academics, they engage directly with dominant actors and policy processes to tackle injustice and risks to sustainability in specific socio-environmental contexts. For us, the social agency of CAI and their praxis hold significant promise for profound change in the system, as they work not in isolation, but rather within and beyond the institutional and discursive regularities of environmental fields. They are also driven by concerns for injustice and hence engaged in building wider alliances for change with policy makers, activists and academics. We take this understanding to show the importance of agency for transformation, without losing sight of how CAI are always situated within wider networks and politics that enable (or sabotage) their efforts. We argue that CAI praxis in environmental fields provides a unique theoretical lens to deepen our understanding of the ‘how’ of transformation.

The motivation for our analysis comes from the absence of conceptually articulated, practice-based theorizing on transformational change. This work evolved from our critical action research on community-based forest governance in Nepal, where some of us were actively engaged as CAI, exposing the hegemony of techno-bureaucratic practices and working to empower disadvantaged communities (Ojha 2006, 2013; Fischer 2017). We draw on extended periods of research by the authors − 30 years in Nepal, 19 years in Central America, and 30 years in Kenya. The three cases show the interplay among intellectual action, the politics of knowledge, and community engagement in environmental governance. Through them, we advance practice-based theorizing of how CAI emerge in relation to these socio-environmental contexts to support transformative change. We believe that this theoretical approach has the possibility to expand current understandings of transformation in ways that are complementary to social movements, macroeconomic processes or technological breakthroughs.

## Transformation: agency, fields, and praxis

The burgeoning research on transformation has exposed a host of dilemmas around the interface between deliberate human agency and structure (Feola 2015; Chaffin et al. 2016; Patterson et al. 2017; O’Brien 2018). Feola ‘s (2015) authoritative review shows that transformation is conceptualised both as an emergent (linked to structures), and deliberate process (linked to human agency). Butzer (2012) advocates shifting attention away from a narrow focus on transformation as unintentional waves of structural change, to deliberate attempts by human agents to shape change. Deliberate transformation in turn, highlights the importance of moments that place individual and collective action in dynamic relation to structural frames: behavioural shifts, organisational forms, and cultural change (O’Brien 2012). Along with Fazey et al. (2018a), our work here draws attention to the role of praxis and agency as relational and cross-scalar processes that help explain possibilities for transformative change. While Fazey et al. (2018b) focus mainly on structural and collective practices, they nevertheless recognise the importance of individual agency in sparking change. We are especially curious about how human agency can be a trigger of transformative change (O’Brien 2012; Otto et al. 2020), but in conjunction with structure and focussing on critical intellectual action.

Critical intellectual action that challenges the status quo has long been recognised as triggering social change: examples include decolonisation (Said 1979), critical scholarship (Gramsci 2000; Hall 2016), deconstructive approaches (Escobar 1995) and feminist theory (Haraway 1991). Our aim here is to advance an approach that places the knowledge-centric practices of individuals within specific fields of power (Bourdieu 1984), which co-evolve with the agency of those who attempt change in the system. This approach builds on a body of environmental governance work that investigates the agency-structure interface using the lenses of subjectivity and symbolic violence (Ojha et al. 2009; Nightingale 2011; Nightingale and Ojha 2013), Bourdieu’s fields and deliberative politics (Ryfe 2007; Ojha 2008; Ojha et al. 2014), critical action research (Ojha 2013), and the political nature of climate adaptation (Eriksen et al. 2015). This strand of literature has established that reproduction and transformation in social practice is strongly linked to critical agency and culturally and politically embedded relationships. It reinforces the view that the potential for long-term profound change exists in the dynamic interface between individuals and the structures of power and cultural regularities (field). We thus approach change from both sides (agency and structure), believing that individuals as social agents can be important catalysts, but also that they need to work within enabling contexts and in alliance with other agents of change in a dynamic social universe.

To link agency with the politics of knowledge and social action, we use Bourdieu’s conceptualisation of the ‘field’ to capture interwoven social relations, political economies and cultural norms (Bourdieu 1984, 1998) that constitute domains like environmental governance (Ojha 2008; McDonald 2016). We believe that the ‘how’ question on transformation can be tackled by exploring these recursive dynamics between agency and structure (field) by taking a more optimistic view than Bourdieu (1984) himself of the deliberate agency of social actors. Bourdieu’s notion of ‘field’ characterizes how actors engage in a complex social universe to contest and access different resources or capitals, and in the process, how they take different positions and develop differentiated dispositions to those social practices (Bourdieu 1984; Ojha 2008). Social fields are ‘‘major areas of practice” sufficiently distinct from each other (Bourdieu 1984), such as ‘‘sport, music, food, decoration, politics, and language” (Bourdieu 1984: 208). We mobilise Bourdieu’s fields as relatively structured settings, but also constantly shifting and contested social arenas in which a set of human dispositions co-evolve with the field (Swartz 1997: 117). The field provides a dynamic setting for access to, control over, knowledge of and distribution of resources (Rocheleau et al. 1996) as well as struggles over social difference that shape development (Nightingale and Ojha 2013).

Analyzing CAI praxis in relation to fields allows us to explore the ‘how’ question of transformation that has too often fallen in the cracks between theory and practice (Vogel and O’Brien 2021). From this lens, transformative change is about the co-evolution of agents and the reshaping of the field through crisis, praxis, and contestations. Central to these dynamics is the interplay between the intellectual actions of CAI and various forms of dissonance in the field. CAI are of course not a panacea for transformation—they are themselves situated in uneven political fields and are relatively privileged—yet what interests us is the potential of their praxis to influence and spark political change. The focus on CAI praxis offers a nuanced and situated analysis of agency and structure to understand the systemic change underpinning transformation. The three cases provide a repertoire of evidence, showing the links between the social agency of CAI, the politics of knowledge, coalitions for change, and likely trajectories of transformation. Our cases feature various forms of transformational change which CAI praxis supported: strong community rights backed by legislation in Nepal, community access to land and forest resources in Kenya, and indigenous people’s recognition and access to land in Central America. While the cases do not offer complete answers, they do illustrate some powerful insights into transformative change in environmental governance.

## Shifting state-controlled forest regime to community forestry in Nepal

### The field of forest governance

Nepal’s forests have long been a crucible of political action (Malla 2001; Shrestha 2001; Khatri 2018; Nightingale et al. 2018), and as such, are part of the highly contested field of environmental governance (Ojha 2013). In the 1950s, the government nationalised forests, alienating traditional rights of local communities, which created mistrust between people and the state, and caused deforestation. By the 1970s global attention to the degradation of Nepal’s forests catalysed coalitions of national and international actors to imagine a new system based on community management, rather than government control (Gilmour and Fisher 1992; Ives and Messerli 1989). After the 1990 democratic revolution, the community forestry system became a keystone of grassroots activism and local democracy (Pokharel et al. 2007; Karna et al. 2010; Ojha 2014; Bijaya et al. 2016). Of interest to us here is the emergence of CAI within the highly technocratic field of forestry (Ojha 2006) who succeeded in spearheading major changes in forest governance. These included the first generation of reformist forest officials and action researchers working with bilateral development projects from the 1970s to the early 1990s, followed by a second generation of researchers working in NGOs.

### CAI and their praxis

The first wave of profound change took place in the 1970s, when a few government forest officials working at the district level experimented with allowing communities to participate in creating forest management rules and to access forest products as usufruct rights. Perhaps the first was TBS Mahat, a widely respected CAI who pioneered community forestry from within the government system at a time when the policy insisted upon centralised control (Mahat et al. 1986). In an interview with us, Mahat described how as a District Forest Officer, he spent a lot of time walking the hills, and hearing about community issues first-hand. He became very concerned by the wide-spread degraded forest areas and the difficulties people faced in meeting their fuelwood requirements. After fruitless efforts petitioning the central government for more reforestation funds, Mahat focused on the willingness of local people to collaborate. He described how he capitalised on a legal loophole to justify the formation of local user committees based on the local (*Panchayat*) government laws (at the time, there was no specific law to allow communities to access ‘national forests’): “In my job my interest was, or my politics were, to put forestry as a development agenda at local level,” (interview 26 December 2017).

He went on to describe in detail a disciplinary meeting at the Forest Department in Kathmandu. He knew his work was risky, so he had his resignation letter inside his coat pocket alongside a written statement justifying how he interpreted the law. Fortunately, he was able to convince his superiors of the merits of his experiment, helped by a parallel process initiated by international donors. The ability of Mahat and his colleagues to critically reflect on existing practices and to recognise the importance of transcending policy triggered major transformations in how forest governance policy evolved over the next 40 years.

Nepali officials like Mahat were joined by foreign researchers in donor projects responding to the global outcry over the so-called Himalayan land degradation crisis (Eckhom 1976). The Nepal Australia Forestry Project began in the late 1970s, which brought curious action researchers such as Don Gilmour, who stepped back from their formal organisational mandate to ask critical questions about why things were not working well (Gilmour and Fisher 1992). As Gilmour recounted, his policy-oriented work in the 80 s in Nepal was not actually explicitly supported by his seniors:

I was told explicitly that “…we were here (in Nepal) for technical knowledge  transfer … related to nursery techniques, plantation establishment, silvicultural practices, etc. I was instructed not to get involved in policy matters, as that was something for the Nepal government (Interview March 2021).

Both international forestry experts and Nepalese officials continued to reshape the field of forest governance. All were clear in interviews with us that it was both their intellectual capability and on-the-ground experience of governing forests that triggered their desire to take professional risks against the status quo. These pioneering efforts on decentralising forest rights were joined by Nepali CAI such as Narayan Kaji Shrestha who initially worked for donor funded projects and later became active as a civil society activist pushing community-based forestry.

These pioneers worked in tandem to persuade the Forest Department to allow community groups to have rights of access to forest areas, and created functioning models of community forest management across a wide swath of the Middle Hills region. First-generation CAI sparked the formulation of a new Forest Act 1993 (and its by-laws in 1996). This law was among the world’s most progressive, recognising community rights over government forest areas. As a result, by the late 1990s, the number of community forestry groups multiplied exponentially across the country, establishing community forestry as one of the most important ‘development successes’ in Nepal.

A second generation of CAI emerged after the 1993 Forest Act, inspired by wider democractic political reforms. The number of CAI multipled and their work supported change through critical research, empowerment of community groups, and faciltiating dialogues between community leaders and the government. Some worked with donor projects to demonstrate the impact of community participation on forest cover (Branney and Yadam 1998), while others working from within the government highlighted the economic potential of community forestry (Kanel 2008). NGO based CAI provided critical insights and training to community leaders which helped to shift it from a donor initiative to one driven by social activists (Shrestha and Britt 1997; Timsina 2003; Ojha 2009). A coalition of CAI working across donor projects and civil society organisations (most prominently Kaji Shrestha) saw the need to support community forestry groups to come together as a federation so that they could have a collective voice against threats of centralisation. In parallel, politically engaged community leaders joined the community forestry movement across the country, and the Federation of Community Forestry User Groups-Nepal (FECOFUN) emerged in 1996 and quickly became the country’s largest civil society network and a key partner of CAI (Ojha et al. 2012). Numerous government foresters embarked on post-graduate studies abroad and came back to the country, most undertaking research work on community-based forestry. CAI such as Kaji Shrestha have the capability to offer ‘reorientation trainings’ to officials on participatory forestry (Gronow and Shrestha 1990).

However, underneath the layers of progressive change and community empowerment, this story has a bleak side too. Changes in the organisational culture of the forest department have been extremely slow. The bureaucracy has never fully accepted the rights of communities, or certainly not to the extent that the 1993 Forest Act provided (nearly complete autonomy once a forest area was handed over), something which persists as of this writing. In the early 2000s, a new generation of trained forestry and environment intellectuals entered the field. Perhaps, most prominent were those who created ForestAction Nepal (FAN) in 2000 (the lead author of this paper is a co-founder). FAN quickly became a key institutional home of CAI wherein they: created significant space for research, policy engagement and the dissemination of critical knowledge by launching a practitioner-oriented research journal and a policy briefs series; experimented with various forms of stakeholder dialogues; and engaged in action research. FAN worked closely with FECOFUN and reformist forest officials to help cement community forestry as the main instrument of forest governance in the hill districts (Kanel and Acharya 2008). For example, FAN created a policy learning group of forest officials, FECOFUN leaders, and forestry experts to accelerate the implementation of the community forestry program across the country. The repeated efforts from within the government to rewrite the law or curtail community autonomy (Shrestha 2001; Ojha 2008; Britt 2001; Sunam and Paudel 2012) became central to FAN’s research, community empowerment and policy dialogues, to push against the risks of re-centralization (Sunam et al. 2013; Basnyat et al 2020).

An example of FAN’s transformative work was about preventing a regressive change in the 1993 Forest Act during 2011–2012. The Government drafted a bill to amend the fundamental rights of Community Forest User Groups (CFUGs). FAN undertook a quick and strategic assessment of this proposal and published a Policy Note (Paudel et al. 2012) which outlined the likely consequences of the policy change. The Note, for example, highlighted: “…The increased bureaucratic power and weakening of group autonomy as proposed by the amendment may have four foreseeable consequences…”. Among these consequences, as the Note identified, was the executive committees of CFUGs becoming accountable to the government forest office rather than the CFUG assembly. FAN’s analysis, just at the time of peak debate, on the law amendment bill had two immediate outcomes. First, FECOFUN picked up the messages for its nation-wide campaign for community rights, building up political pressure against the planned amendment. Second, the government and parliamentary groups invited FAN-based CAI to share their recommendations, eventually leading the government to withdraw the Bill and engage in open consultation for the best policy direction.

### Reshaping the field

Over two generations, CAI praxis has profoundly shifted the logic of forest governance in Nepal towards community-based systems and helped sustain it against efforts at techno-bureaucratic recentralisation. CAI in Nepal have also contributed to the theory of critical action research and deliberative practice as well as methodological tools such as policy labs that bring together contesting stakeholders in co-producing policy solutions (Ojha et al. 2020). CAI have never worked alone, but rather sought partnerships with local communities, national government officials, and global knowledge partners. Yet, their agency has been crucial. Building from their overseas education and networks, second generation CAI’ innovations with action research and their ability to publish their work in global academic outlets provided symbolic power to influence change.

CAI praxis and insistence upon multi-stakeholder policy dialogues, informed by the complex politics of multi-scale forest governance, have challenged the binary of ‘state versus community’ within policy formulation (Banjade et al. 2007; Paudel et al. 2008; Khatri 2012; Ojha 2014). Unlike the advocacy of community rights work done by FECOFUN, CAI built wider knowledge partnerships with critical and action-oriented scholars such as Nightingale (Nightingale 2002; Nightingale and Ojha 2013), McDougall and Prabhu (Fisher et al. 2007; McDougall et al 2008), Cameron (Cameron and Ojha 2007), and Hall (Ojha and Hall 2021) to expose the limits of community institutions and explore the prospect of systemic change (Luintel 2006). Rather than relying on mere political negotiation of rules and practices, this second generation of CAI insisted on the importance of research-informed dialogues wherein diverse actors came together to reframe authority in the forestry sector. Second and third generation CAI are now actively engaged in shaping climate policies and adaptation responses, especially in relation to forests and community forestry user-groups (Khatri 2018), as Nepal embraces a new federal governance system and formulates new environmental policies for its commitments under the Paris Agreement. For us, this is evidence of how CAI both emerged from the field, and also contributed to reshaping it in a transformative manner.

## Reworking colonial socio-environmental relations in Central America

### The field of environmental governance

In Central America, indigenous land rights have been at the heart of socio-environmental struggles for hundreds of years (García Babini 2012; Hurtado-Paz y Paz 2019; Wainwright and Bryan 2009; Ybarra 2018). Guatemala and Nicaragua are mired in increasing authoritarianism but struggles against this have triggered a movement for agro-environmental justice, indigenous autonomy (Grandia 2020) and legal recognition. While certainly not the sole force, CAI have played crucial roles in transformative processes, resulting, even if sometimes temporarily, in the safeguarding of community rights over environmental resources. Below we illustrate how their approach has developed new analytics and strategies informed by research-based knowledge as well as practical action aimed at removing injustice. CAI work has been driven by a high degree of commitment to justice for marginalised groups sometimes at the cost of their own lives.

In Guatemala and Nicaragua, colonial legacies are related not only to the countries’ former occupation by Europeans, but also to the colonisation by capitalist modes of agricultural production that are founded in non-indigenous ways of relating to land and nature (Offen 2003; Grandia 2012). The environmental governance field where CAI emerged are rife with struggles over recognition of indigenous cultures and identities and land. For example, Maya indigenous populations in Guatemala have faced systematic discrimination since the Spanish colonization (1524–1821). More recently, agrarian extractivism through the promotion of large-scale plantations has further alienated their rights to land (Alonso-Fradejas 2012). Several lawyers and intellectually engaged development activists have worked with these communities to strengthen resistance strategies, for example by supporting local communities in the defence of their territories through the (re-)construction of collective land-based identities (Alonso-Fradejas 2015). These CAI have also advanced counter hegemonic concepts like food sovereignty which, as envisaged by the Mayas, is both a means of fighting against extractivism and advancing a life-transformative project (Alonso-Fradejas 2015).

Likewise, indigenous people in Nicaragua face similar problems, prompting groups of CAI to confront these through intellectually engaged and socially grounded actions. Formerly occupied by the British (1655–1859), Eastern Nicaragua became one of the first regions in the Americas in 1987 to establish autonomous polities that guaranteed indigenous and Black communities’ participation in a multicultural government. From the 1990s onwards, this autonomous status has been contested by the Nicaraguan state, claiming the region consists of “national lands” (Wainwright and Bryan 2009).

### CAI and their praxis

Similar to Nepal, empirical stories about socio-environmental struggles in the Central American countries of Guatemala and Nicaragua elucidate the intellectual-practical agency of CAI who played key roles in transformation. While CAI’ actions in Central America can be broadly situated in the field of natural resources management, they are at the same time associated with wider political struggles for justice, equity and democracy. CAI included lawyers and development practitioners who mobilised critical research capacity and coalition-building strategies to safeguard community rights over environmental resources in politically contested environments. These CAI have emerged from the humanitarian, legal and academic as well as development sectors, similar to the Nepal case study. In particular, this example shows how CAI mobilise critical knowledge to challenge hegemonic knowledge claims, and nurture alternative knowledge systems that can be instrumental for transformational processes. While the positive outcomes of such efforts have sometimes been co-opted or even reversed, CAI’ efforts have created important, sometimes worldwide precedents that inspire others in the field.

Within this field of agro-environmental governance dominated by colonial legacies, CAI’ ability to envision and promote alternative and decolonial knowledges (Waziyatawin 2004; Grosfoguel 2007) and practices have been key. In Nicaragua, the Mayangna indigenous community of Awas Tingni sued the Nicaraguan state in 1997 for granting a logging concession to private interests in the Mayangna traditional territory. A CAI who is also a female indigenous lawyer has been working closely with the community from 1996 onwards, and was instrumental to a landmark Human Rights ruling by the Inter-American Court of Human Rights in 2001 that became a precedent for all indigenous groups of the world (Gómez Isa 2017). The ruling recognised that the government had violated the rights of indigenous people, and established, for the first time in history, indigenous communities’ right to their collective land as a basic human right (Inter-American Court of Human Rights 2001).

Besides such higher scale engagement in the environmental governance field, CAI’ partnership with local indigenous communities in Nicaragua has also aimed at demonstrating ways of working with ethnic minorities that overturn conventional, hierarchical ways. In the Nicaraguan case of Awas Tingni, the above-mentioned CAI collaborated with the indigenous community in a novel way and used alternative methods of articulating knowledge systems for achieving transformation in practice. For example, the CAI could have legally represented the indigenous community at the court. Instead, she supported the indigenous community to represent itself through twenty-five of its members. What was at stake was not only winning the case, but also showcasing decolonised ways of working together. This further showed the world that indigenous groups have legitimate and established ways to ensure justice and organizational systems to deliver it in practice. The CAI explained:

(…) the Interamerican convention of Human Rights does not recognize collective property, it only recognizes private property (…) but we decided to educate that tribunal [the Interamerican Court of Human Rights]. (…) we decided that I would not represent [legally] the community and I would be in the audience as an expert witness. We thought about (…) showing how justice is administrated from the indigenous perspective. (…) if the court did not understand us, how would it understand that for the indigenous community there is not only a material relation with the land but also a spiritual relation? (…) we wanted to connect the right to life to the right to land” (Interview, 13 May 2020 via videoconference).

In addition to the mobilisation of a justice and recognition narrative such as above, CAI have also used technical tools to generate alternative data to empower the voices of marginalised groups. While this type of effort led by CAI may not change all the historically and culturally embedded colonial practices that systematically disadvantage indigenous people, they envision practical pathways for socio-environmental transformations that can serve as benchmarks.

CAI’ capacities to mobilise technical knowledge typically used to serve the interests of the powerful have been instrumental in the defence of indigenous people’s right to land in Central America. CAI in Guatemala have assisted indigenous communities to defend their land rights in the face of increasing oil palm plantations by using GPS to map community rights, but in accordance with their indigenous vision, and in a participatory way. Such maps helped to materialise the traditional system of land rights and to establish community land management plans, thereby empowering the community to develop counter propositions for the Guatemalan state and its World Bank funded land legalisation programme that did not recognise indigenous community rights (Dietsch et al. 2014). Similarly, in the Nicaraguan Chorotega indigenous communities, one of the co-authors of this article supported the creation of 3D participatory maps, which encouraged critical dialogues between indigenous and non-indigenous populations about rights, obligations and conflicts (Gonda and Pommier 2008).

### Reshaping the field

The Central American case further shows how CAI’ engagement alongside the indigenous cause has been informed by a strategy for embracing uncertainty. A Nicaraguan female CAI, another lawyer, reflected on how she chooses to focus on the processes rather than ideal results that are often highly unpredictable in a context of increasing authoritarianism. She used the example of the draft of Law 445 on Indigenous Lands in Nicaragua (National Assembly of Nicaragua 2003) that she herself helped formulate, whilst acknowledging that it might never be approved. Like the promotion of academic-practitioner journals and policy briefs in Nepal, she believed that creating and circulating such drafts are an important part of building pressures for transformational change. To her surprise, the draft Law was recently approved without modifications (Interview, 15 June 2020 via videoconference). Here, she was focusing on the process of transformation, even acknowledging the unlikeliness of this being achieved, rather than the uncertainty. In that sense, CAI’ ability to recognize and interpret injustices, act during uncertainty and political turmoil, as well as engage with hegemonic politics appears to be a core part of the democratic transformative practices they support.

The Central American example thus illustrates how CAI’ actions are situated within long-term dynamics of the environmental field, focussed on efforts to create enduring examples, and are about reworking social relations rather than trying to reverse them (Wainwright and Bryan 2009). In other words, CAI’ works are not just about changing power relations at a certain time in a certain place; rather, it is about changing the cultural, social and political economy of the field that created those power relations in the first place.

## Influencing forest and climate change policies in Kenya

### The field of environmental governance

Like the other two cases, Kenya has struggled to transform relations over land and natural resources since independence in 1963, creating a fertile field for CAI to emerge. During the colonial era in Kenya, environmental governance became entangled with social and political injustices. Large tracts of land were concentrated in the hands of a few wealthy landowners, while many smallholders and pastoralists were displaced to marginal lands. Forests came under the ownership of the government, legally excluding local populations (Cavanagh 2017; Eriksen et al. 2006). As result, environmental governance remains a site where political contestation and competition play out in Kenya, with efforts to formalise local community resource rights occurring amidst increased arming of local populations (to protect against poachers and cattle raids), and where oil development relies on private and public security (Shilling et al^2015^; Pellis et al. 2018; Muok et al. 2021). Transformations in environmental governance are long term processes, and partial at best, as local resource rights have become embroiled in the political economy of oil exploitation and national political rivalries. Like Nepal and Central America, several generations of CAI have emerged in this shifting field to change discourses and promote alternative knowledges of environmental change. One of the authors of this paper represents the second generation of CAI, working in the academic-political space opened up by the first generation of CAI from the late 70 s onwards.

While the Kenyan environmental governance field has gone through major shifts since independence, these transformations have taken time to manifest in legal frameworks and local level practice. They can best be described as partial, turbulent and contradictory. Regaining rights to land that had been given to white settlers was a key motivation in the Kenyan independence movement, yet forests and drylands remained under government ownership and large private farms were, in part, redistributed to a new Kenyan political and economic elite rather than to smallholders and pastoralists. Land quickly became a currency for securing political support from particular ethnic groups, spurring land clashes and dispossession (Anderson 1987; Anderson and Lochery 2008). At the same time, there was continuous pressure to shift resource control back to local populations.

### CAI and their praxis

A first wave of CAI engagement in Kenya explicitly linked ideas of social justice and environmental sustainability—fast rising on the international agenda—with the rights of disadvantaged communities. Several CAI combined academic positions with civil society action, mobilising their authority as intellectuals as well as legitimacy in the local population to articulate agendas for change. For example, Professor Wangari Maathai’s Nobel prize-winning work caught the world’s attention by initiating the Green Belt Movement in 1977, a response to environmental degradation, linking local women’s empowerment with environmental stewardship and tree planting (Maathai 2008). Yet space for transforming policy was confined by the restrictive political system, and Maathai herself spent several periods in jail due to her political engagements. She nevertheless went on to become cabinet minister for environment from 2003 to 2005 where she spearheaded the Forest Act of 2005. This Act was the first substantial revision to forest law after independence and the first legal instrument to recognize the role of communities in forest management, ushering in a paradigm shift towards participatory forest management, a decade after Nepal had enacted such legislation in 1993.

The efforts of CAI like Maathai worked to shift policy discourses, putting environmental sustainability, the empowerment of women, and the resource rights of local populations on the political agenda. This laid a foundation for significant shifts in the field after 2000. Activist academics concerned with injustices in resource governance drew on ground-breaking research and international PhDs to insert social justice, community resource rights, and environmental sustainability into policy debates. Community rights recognised by forest policies were formalised in the Land Policy (2009) and the Community Land Act (2016). In 2013, newly constituted County level authorities were given financial resources and some decision-making authority in the management of forests, water and land (the national government retained ownership of these resources) (Cooke et al. 2016). The actions of several other CAI during the 1980s and 1990s was pivotal to these transformational changes. Notable ones include Professor Calestous Juma’s work on biodiversity and justice “The Gene Hunters” (Juma 1989), and the independent, critically engaged research organization he established. He put biodiversity and biotechnology on the policy agenda, contributing momentum towards the formulation of the National Biotechnology and Biosafety Policy of 2009 and the Biosafety Act of 2009. Similarly, Professor HWO Okoth-Ogendo's advocacy-based research on African indigenous land rights revolutionized land reform in several African countries (Okoth-Ogendo 1991). This engaged praxis of Juma and Okoth-Ogendo linked research with policy deliberation and writing processes. Together these three CAI (and others) helped confront colonial framings of environmental governance persistently disadvantaging local Kenyan populations. This first-generation CAI prepared the ground for the engagement of a second wave of CAI to contribute to policy shifts that only became possible once multi-party democracy widened the space for political deliberations.

Despite these progressive changes, this story is also one of long struggle and partial success. Both Okoth-Ogendo and Calestous Juma were very conscious of the social acceptability of their work, especially in the eyes of the Kenyan authorities. Juma had to balance good relations with international donors and avoid openly challenging the Moi regime. Maathai was criticised for her policies when she became Kenya’s environment minister. Later, as Harvard professor in the US, Juma was heavily criticised when he supported genetic engineering at the behest of business-giant Monsanto (Adenie 2018). However, it should be noted that these contradictions surfaced when they had taken on new roles and were not directly engaged in the field. Likewise, changes catalyzed by CAI praxis are slow to produce results. Community ownership of land was not recognized until 2016 (Republic of Kenya 2016), and County governments are still struggling to assert their authority. The Community Land Act 2016 reinforces territorialisation and ongoing political competition, and excludes those unable to claim community belonging (and hence land rights). The increased focus on community rights in environmental stewardship is challenged by increasing oil exploitation in the drylands and militarization of authority, as well as climate change adaptation planning (Omukuti 2020; Ng’ang’a and Crane 2020; Lind 2018). Transformative changes thus represent the culmination of decades of CAI engagement in political struggles, often with incomplete outcomes.

### Reshaping the field

In this shifting field, a second generation of CAI has emerged. International commitments to the Paris Agreement and the corresponding plethora of adaptation policies and plans are emerging as a new field of praxis. The Kenyan co-author of this paper has been a member of the climate change policy working group through which these new policy and legal documents were delivered. He was also lead author of the controversial Turkana Climate Change Policy, Climate Bill and Climate Change Finance Regulation, which prioritises green energy. These challenged fossil fuel based economic development which has been backed by vested national and international government and business interests, both of which have a strong voice in policy deliberations. Local populations, in contrast, contest—sometimes violently—the loss of control over land and little local employment or other benefits (Lind, 2018).

To negotiate this highly contested field of engagement, a team of CAI joined forces with non-government organizations through the umbrella of the Climate Action Network (CAN) International to influence Turkana policy. The CAI leveraged their research backgrounds (Muok and Kingiri 2015), senior academic positions, and partnerships with civil society to partially shift the discourse about appropriate development. Their engagement spanned from local communities to the global sphere: Kenya’s international commitments to the UN Framework Convention on Climate Change to reduce emissions under the current nationally determined contribution (NDC) proved instrumental. Policy deliberations emphasised that a transition to clean energy sources is inevitable and must be implemented in a just manner. It remains to be seen how community land rights and energy development are negotiated in practice through formal and informal decision-making, especially when dispossession, marginalization and violent capture of resources by the elite remain rife (Cavanagh 2017).

The case of Kenya illustrates how CAI are nested in the field of existing power and knowledge relations, yet actively draw on civil society’s ability to amplify messages, penetrate state systems as experts, influence global knowledge domains, and use symbolic power to directly contest particular aspects of socially unjust relations over natural resources. Such efforts of CAI in environmental governance are not the sole force behind transformation, but arguably pivotal. The extent to which CAI can contest and resist being enrolled in dominant discourses and asymmetric power relations between the political intellectual elite and local populations is key to the transformative potential of their praxis, even if only partially successful. The case nevertheless suggests that by grounding their work in a clear justice and ethics position and by fostering partnerships between academic actors, civil society and local populations, including between the global South and the North, CAI can mobilize alternative knowledges and contribute, over time, to transforming sustainability agendas and environmental governance.

## Discussion: CAI and the prospect of transformation

Transformation does not result from just one factor, process, or even a political act. Our focus in this article is on exploring the prospect of transformation through critical intellectual practice in fields of environmental governance. Extending the debate around the ‘how’ of transformation, our work indicates the potential of intellectual action in transformational change, which varies according to the realities of environmental governance fields. Focussing on the dynamic interface between CAI praxis and the field, here we advance theoretical reflections on four key aspects: the dynamics of CAI praxis, how CAI praxis can reshape the field, temporality of transformation, and areas for future research.

### CAI’ praxis

CAI praxis has emphasised breaking the nexus between knowledge and power asymmetries through inquiry-based and action strategies. On the inquiry front, CAI have been sceptical of the academic obsession with theoretical knowledge (Bourdieu 1990) and responded to the call for politically engaged scholarship (Hale 2008), to tackle real-world problems faced by communities. On the action front, a common attribute of CAI is their intellectual motivation driven by social and environmental problems affecting the wider community. In our cases, these motivations were animated by the desire to overcome colonial control of natural resources in Kenya and Central America, and state centralization of forest governance in Nepal. Such a strategy of using research simultaneously for knowledge *and* action differentiate CAI from other scholars or activists.

CAI begin by breaking away from common-sensical knowledge held by communities, before embarking on empowerment, and critical self-reflection. They believe community empowerment is possible only when they create an ‘epistemological break with the primary experience’ as suggested by Bourdieu (Bourdieu et al. 1991, p6). This means CAI rarely start by waiting for their ‘clients’ or ‘beneficiaries’ to ask for a ‘research service’—instead they proactively reach out to communities and authorities and initiate critical conversations. Their closest allies are the marginalised groups who, in dialogue with CAI, question the hegemonic power in environmental governance [see also Kincheloe and McLaren (2000)]. Such alliances animate communities to challenge hegemonic framings and articulate new solutions to socio-environmental problems. Alongside these deliberative practices (Forrester 1999; Dryzek 2006), CAI also cultivate a habit of critical self-reflection to challenge their own frames and assumptions, much like the way Schon imagines ‘reflections-in-action’ (2008). These moments of reflection are vital for transformation, and indeed, it is when people stop being self-reflective that their legitimacy and efficacy as CAI declines.

Further, as our cases show, CAI’ critical inquiry has exposed historical and political roots of knowledge and power hegemonies that drive social marginalisation and risks to sustainability. Their critical epistemological work has involved creating new evidence to inform both academic and policy debates and to articulate alternative ideas of governance. In situations of controversy and deadlock in policy debates, CAI have opened spaces for negotiation and participatory experimentation with new ideas. Through all these tactics—from injecting critical evidence to using legal instruments—CAI have enhanced the quality of policy deliberation in times of heightened policy contestation.

### Reshaping the field through CAI praxis

A large body of social theory exists to support the view that systems of governance and underlying social norms rarely evolve without co-evolutionary links between the critical consciousness of social actors and wider structures (Giddens 1984; Bourdieu 1998; Haraway 1991). Advancing this theoretical front, we show that the interface between CAI (agency) and environmental governance fields (structure) offers an important conceptual tool to understand transformation.

First, the prospect of transformation is linked to the interface between CAI praxis and the field. While CAI themselves are relationally co-produced with the socio-environmental realities of the field, their praxis has enabled them to identify, recognise, and challenge the underlying logic of environmental governance fields. The existence of deeply rooted institutions of power means transformational change rarely happens without a deliberate disruption to the unchallenged rules of the field. In fact, it is primarily through the ways in which CAI are able to disrupt the dynamics of the field that the transformational edge of CAI praxis becomes clear. Second, opportune moments targeted by CAI emerge when the field periodically experiences crisis. In all the cases, we find that transformation in power asymmetries requires constant work, for example to defend the hard-won autonomy for community management when it is challenged by a new environmental policy. Such an agile response to crisis in the field by CAI is possible through ‘real-time CAI praxis’.

Third, CAI’ praxis itself rests, to a significant degree, on the logic of the knowledge economy which underpins a field of governance. Western university degrees, publishing in the global scientific media and building transnational partnerships are among the tactics that have proven successful. CAI confront not just intersubjective situations of power and knowledge relations, but also the underlying economy of cultural and political resources which support specific practices of governance. Fourth, when the dominant players in the field find it difficult to ignore the voices of marginalised communities or critical arguments of CAI, they invite CAI and community leaders to contribute to policy. In such situations, CAI praxis then also includes working from within the structure of governance to change its underlying logic, as found in the case of Nepal and Kenya.

Finally, the field is a multi-scalar reality and so is CAI’ praxis, and this multi-scalar interface has been crucial in transformative change. CAI have targeted the knowledge-power nexus, sparing no authorities or structures that have contributed to problematic situations. Similarly, CAI have engaged with formal politics to tackle multi-scalar dynamics, as in the case of Kenya where some CAI have moved between academia, politics, and action. Beyond the national borders, international agendas such as the Paris Climate Agreement have created new challenges—and opportunities for CAI praxis across scale. In all these dynamics, there are possibilities for transformation or simply the reinforcement of the status quo, a point we now turn to in relation to temporality and transformation.

### Temporality

CAI praxis and its interface with the field illuminate the complex temporal aspects of transformation. As transformation is about profound change in systems and behaviours, effective CAI praxis is dependent upon long-term engagement. CAI-initiated change is enduring but slow, taking two generations to shift state control over resources to local communities in all three cases, albeit with somewhat different outcomes. In the Nepal and Kenya cases, at least two generations of CAI have worked on similar agendas: community-based forest governance in Nepal and land rights and inclusive natural resources management in Kenya. In both cases, the first-generation CAI contributed to environmental policy reforms, while the second generation engaged in defending or refining those policies as they were implemented. With climate change and other new policy agendas, a new generation of CAI are emerging to ensure community rights are protected. While CAI come and go as part of the natural cycles of life and careers, the environmental fields remain to give rise to new generations of CAI when the old ones retire, or lose their critical edge.

### Future research

Our cases suggest two areas of future research around the role of intellectual action in transformation. First, how can critical intellectual action be sufficiently amplified to address the dynamic realities of environmental governance fields? Without sustained engagement and without new generations stepping into spaces that have been created, or are under threat, transformational gains can be rapidly undone. This is important because each of these stories has a dark side, pointing to the limitation of CAI, both in relation to their capacity to tackle highly entrenched power relations, and the rapidly changing political economies of symbolic and material resources in the field affecting their survival and work. What dynamics of the field and strategies of CAI praxis support transformative change in governance emerge as an important area for further research.

Second, closely related to the above point, what makes CAI praxis thrive in adverse political and economic environments? In the Central American case, some CAI have literally lost their lives while defending indigenous struggles, while others maintain anonymity for fear of repression. In a subtle example of adversity, many CAI in Nepal now struggle to find the personal and professional space to engage in the critical action research that built their reputations, and rather are hostage to ‘research for development’ contracts. As a result, their praxis is reshaping discourse and power on some fronts, and also serving to simultaneously entrench them on others (Nightingale 2018; Ojha 2013). In all three cases, CAI find themselves needing to play the political game adequately to maintain any operating space at all which can look contradictory and undermine the power of research-based work. These findings raise theoretical questions about how CAI and their allies can both gain adequate power, first to thrive in adverse contexts, and then to challenge dominant authorities.

## Conclusion

Research on transformation has traced its causal drivers in politics, technological breakthrough, social movements, or macro-economic processes. In contrast, this article brings theoretical insights on how critical and action-oriented intellectual work can trigger transformational change in environmental governance. Drawing on our current and previous research in Nepal, Nicaragua, Guatemala and Kenya, we have illustrated how various types of critical action intellectuals (CAI) emerged and engaged in praxis triggering systemic change in environmental governance. At its core, CAI praxis involves creating alternative and action-oriented knowledge to challenge dominant policy assumptions and empower disadvantaged groups in the processes of governance. CAI praxis is distinguished from other kinds of activism by emphasising the need to change discourses and reframe policy narratives, in addition to working towards concrete changes in access to and control over resources. They thus focus on knowledge reframing *and* on active engagement with local people, policy makers and other communities of practice. While CAI do not work in a vacuum, nor are the sole force of change, we nevertheless show that the praxis of CAI within fields of environmental governance has the potential to trigger transformation.

At least three theoretical insights are noteworthy. First, since systems of governance are rooted in the self-reproducing logic of fields, any attempt to effect systemic change requires exposing the epistemic foundations of the dominant order, such as through utilising the power of critical evidence, social experimentation, and building of critical epistemic communities. CAI have emerged as strong social agents to take on such challenges, and central to their success are their abilities to question existing ways of thinking and promote new ways of acting as well as playing with the underlying and self-reproducing logic of the field with the intention of overturning it. This means that systemic change inevitably requires some form of critical intellectual work that is capable of connecting practice, discourse, and politics within and beyond the boundaries of the field.

Second, the work of CAI is itself relationally co-produced with the dynamics of fields, and hence the transformational potential of CAI praxis is significantly linked to the structural regularities and recurrent moments of crisis in the field. Through CAI praxis, the field is challenged, and when successful, reshaped. That reshaping in turn demands new forms of engagement and reflection by CAI, as realities of the field shift, or previous attempts at change fail. In the long and arduous trajectory of system change, CAI as individuals may change their career or engagements, but the field remains to give birth to a new generation willing to take on the challenge. An important conclusion here is that systemic change emerges not just from the strategic acts of critical action intellectuals, but also from the interplay between CAI praxis and the dynamics of the field, especially when moments of opportunity emerge in the temporal trajectory of the field.

Finally, as transformation involves changes in systems, behaviours and mindsets, no single event or action is likely to bring about such profound change. Systemic change requires continuously pushing the work of knowledge and community mobilisation, roles which CAI take on, with their action oriented epistemic research as well as political will to engage in action for change. However, they are also part of the uneven political field, and their success is not just linked to what they do but also to their identities and positions in the field. CAI praxis is also contingent on the possibility of CAI sustaining their work in challenging times in the field. This means understanding the prospect of CAI praxis has to be contextual and a dynamic process of learning and contextual theorising, locating the prospect of transformation in the interface between CAI and their praxis in the field.
